# Novel oncolytic chimeric orthopoxvirus causes regression of pancreatic cancer xenografts and exhibits abscopal effect at a single low dose

**DOI:** 10.1186/s12967-018-1483-x

**Published:** 2018-04-26

**Authors:** Michael P. O’Leary, Audrey H. Choi, Sang-In Kim, Shyambabu Chaurasiya, Jianming Lu, Anthony K. Park, Yanghee Woo, Susanne G. Warner, Yuman Fong, Nanhai G. Chen

**Affiliations:** 1 Department of Surgery, Division of Surgical Oncology, City of Hope National Medical Center, 1500 Duarte Rd., Duarte, CA 91010 USA; 2 Center for Gene Therapy, Department of Hematologic and Hematopoietic Cell Transplantation, Beckman Research Institute, City of Hope National Medical Center, Duarte, CA 91010 USA; 3 Gene Editing and Viral Vector Core, Beckman Research Institute, City of Hope National Medical Center, Duarte, CA 91010 USA

**Keywords:** Cancer therapy, Immunotherapy, Translational, Vaccinia, Viral vectors

## Abstract

**Background:**

Pancreatic ductal adenocarcinoma (PDAC) has been increasing by 0.5% per year in the United States. PDAC portends a dismal prognosis and novel therapies are needed. This study describes the generation and characterization of a novel oncolytic chimeric orthopoxvirus for the treatment of pancreatic cancer.

**Methods:**

After chimerization and high-throughput screening, CF33 was chosen from 100 new chimeric orthopoxvirus isolates for its ability to kill pancreatic cancer cells. In vitro cytotoxicity was assayed in six pancreatic cancer cell lines. In vivo efficacy and toxicity were evaluated in PANC-1 and MIA PaCa-2 xenograft models.

**Results:**

CF33 caused rapid killing of six pancreatic cancer cells lines in vitro, releasing damage-associated molecular patterns, and regression of PANC-1 injected and non-injected distant xenografts in vivo after a single low intratumoral dose of 10^3^ plaque-forming units. Using luciferase imaging, CF33 was noted to preferentially replicate in tumors which corresponds to the low viral titers found in solid organs.

**Conclusion:**

The low dose of CF33 required to treat pancreatic cancer in this preclinical study may ease the manufacturing and dosing challenges currently facing oncolytic viral therapy.

## Background

New cases of pancreatic ductal adenocarcinoma (PDAC) have been increasing by 0.5% per year in the United States and a dismal mortality rate persists. Currently, PDAC is the fourth leading cause of cancer-related death and is anticipated to be the second leading cause in the United States by 2030. Cancer-related 5-year survival from 1975 to 2008 has had an overall survival increase from 49 to 68%, however, PDAC survival has only increased from 2 to 6% [1]. Surgical resection is the mainstay of curative treatment but as many as 80% of patients present with unresectable regionally advanced or metastatic disease at the time of diagnosis [1]. In the setting of metastatic disease, recent trials using FOLFIRINOX resulted in an incremental improvement of overall survival to 11.1 months compared to 6.8 months seen with gemcitabine [2]. Thus, PDAC will continue to portend a dismal prognosis unless novel therapies are introduced.

Immuno-oncolytic therapy is an exciting area of current cancer research. An enriched understanding of tumor biology and patient immunology has led to the development of the current generation of oncolytic viruses (OVs). Orthopoxviruses, such as vaccinia virus (VACV), have been investigated for their tumoricidal potential in numerous cancers, including pancreatic cancer [3–5]. Characteristics of these viruses that made them favorable candidates as clinical agents include cytoplasmic replication, the excellent safety profile in the global eradication of smallpox, and a short life cycle [6–8]. Clinical trials data from a number of these agents have confirmed a favorable toxicity profile. However, the field would benefit from viruses that are more potent in cancer killing, and that cost less to produce.

The novel oncolytic virus presented in the current study is CF33. It is a chimeric orthopoxvirus selected in a high throughput screen for its efficient viral proliferation and tumoricidal capabilities. In the following studies, we demonstrate the potent ability of this virus and its imaging capable derivative CF33-Fluc, to replicate in and kill a panel of pancreatic cancer cells in vitro. In vivo models of pancreatic cancer xenografts in nude mice were then used to demonstrate the selectivity of this virus for cancer while sparing normal tissues, and to demonstrate the safety of this virus in preclinical models. We also show that treatment of tumors by direct tumor injection can not only produce tumor regression of the injected tumor but also lead to viral spread to distant non-injected tumors and distant tumor regression.

## Methods

### Cell lines and maintenance

African green monkey kidney fibroblasts (CV-1) were purchased from the American Type Culture Collection (ATCC). CV-1 cells were cultured in Dulbecco’s modified Eagle’s medium (DMEM) supplemented with 10% fetal bovine serum (FBS) and 1% antibiotic–antimycotic solution. Human pancreatic cancer cell lines PANC-1, MIA PaCa-2, BxPC-3, SU.86.86 and AsPC-1 were purchased from ATCC. Capan-1 was a kind gift from Dr. T. Ku’s lab (City of Hope, Duarte, CA, USA). All pancreatic cancer cell lines were cultured in Roswell Park Memorial Institute medium (RPMI) 1640 supplemented with 10% FBS and 1% antibiotic–antimycotic solution. Media and supplements were purchased from Corning (Corning, NY). All cells were maintained in a humidified incubator at 37 °C and 5% CO_2_. Cell lines were authenticated by Genetica^®^ DNA Laboratories.

### Viruses

Cowpox virus strain Brighton, raccoonpox virus strain Herman, rabbitpox virus strain Utrecht, VACV strains Western Reserve, International Health Department, Elstree, CL, Lederle-Chorioallantoic (LC) and AS were purchased from ATCC. CV-1 cells were used for both amplification and titration of these orthopoxviruses.

### Chimerization of orthopoxviruses and high-throughput screening

Chimerization of orthopoxviruses was achieved by co-infecting CV-1 cells with all the aforementioned strains of orthopoxvirus. Following the co-infection, one hundred individual plaques were picked and subjected to a total of three rounds of plaque purification in CV-1 cells to obtain 100 clonally purified chimeric orthopoxviruses. High-throughput screening was used to compare the cytotoxic efficacy of these chimeric clones and the parental strains against the NCI-60 panel. CF33 was selected as the chimeric isolate that demonstrated superior cell killing in the NCI-60 panel when compared to all parental viruses and other plaque-purified isolates.

### Generation of recombinant chimeric orthopoxvirus expressing firefly luciferase

To construct thymidine kinase (TK) shuttle vector, the left and right flanking sequences of the *TK* gene were PCR-amplified from CF33 genomic DNA using Q5 High-Fidelity 2X Master Mix (New England Biolabs Inc., Ipswich, MA) and the primers: 5′-GCGCATATGATCTATGGATTACCATG GATGACAACTC-3′ and 5′-CGTTTAACTCGTCTAATTAATTCTGTAC-3′ (left flank), 5′-CAGGTAAAAGTACA GAATTAATTAGACGAGTTAAACGAGC CGTCGACGGATCCGCTAGCGGCCGCGGAGG TAATGATATGTATCAATCGGTGTGTAG-3′ and 5′-GCGGAATTCGTAATTACTTAGTA AATCCGCCGTACTAGG-3′ (right flank). The two fragments were joined together using the method of gene splicing by overlapping extension [9]. The resulting fragment was digested with *Nde*I and *Eco*RI and cloned into plasmid pGPT to yield p33NC-TK. The flanking sequences of *TK* in the shuttle vector were confirmed by sequencing. p33NC-TK contains the left and right flanking sequences of *TK* separated by *Sac*I, *Sal*I, *BamH*I, *Nhe*I and *Not*I, and *Escherichia coli* guanine phosphoribosyltransferase (gpt) gene driven by the VACV early promoter p7.5E as a transient dominant selectable marker.

The Emerald expression cassette with the VACV H5 early/late promoter was PCR-amplified from the plasmid Emerald-pBAD (Addgene, Cambridge, MA) using Q5 High-Fidelity 2X Master Mix (New England Biolabs Inc., Ipswich, MA) and the primers: 5′-GCGAAGCTTGAGCTCAAAAATTGAAAATAAATACAAAGGTTCTTGAGG GTTGTGTTAAATTGAAAGCGAGAAATAATCATAAATAGTCGACCACCATGGTGAGCAAGGGCGAGGAGCTGTTCACC-3′ and 5′-GCGGGATCCATAAAAATT AATTAATCAGTACAGCTCGTCCATGCCGAGAGTGATC-3′. The PCR fragment was digested with *Sac*I and *Bam*HI and cloned into plasmid p33NC-TK to yield p33NCTK-H5-Emerald. The sequence of the Emerald expression cassette was confirmed by sequencing. To generate a shuttle vector containing the firefly luciferase expression cassette with the VACV H5 promoter, the firefly luciferase cDNA was PCR-amplified from the plasmid pCDNA3.1(+)/Luc2 = tdT (Addgene, Cambridge, MA) using Q5 High-Fidelity 2X Master Mix (New England Biolabs Inc., Ipswich, MA) and the primers: 5′-GCGGTCGACCACC ATGGAAGATGCCAAAAACATTAAGAA GGGCCCAGC-3′ and 5′-GCGGGATCCATAAA AATTAATTAATCACACGGCGAT CTTGCCGCCCTTCTTGGCCTTAATGAG-3′. The PCR fragment was digested with *Sal*I and *Bam*HI and cloned into the same-cut p33NCTK-H5-Emerald replacing Emerald to yield p33NCTK-H5-Fluc2. The sequence of the firefly luciferase cDNA was confirmed by sequencing.

CV-1 cells were infected with CF33 at a multiplicity of infection (MOI) of 0.1 for 1 h and then transfected with p33NCTK-H5-Fluc2 by use of jetPRIME in vitro DNA & siRNA transfection reagent (Polyplus-transfection Inc., New York, NY). Two days post infection, infected/transfected cells were harvested and the recombinant viruses were selected and plaque purified as described previously [10].

### Cytotoxicity and viral proliferation assays

PANC-1, MIA PaCa-2, BxPC-3, SU.86.86, AsPC-1 and Capan-1 were seeded in 96 well plates (3 × 10^3^ cells per well) and infected with CF33 or CF33-Fluc at MOIs 1, 0.1, 0.01 in triplicate. A daily cell viability assay was performed by adding 20 μL of CellTiter 96^®^ AQueous One Solution Cell Proliferation Assay (Promega, Madison, WI) to all wells. Absorbance was measured after 1 h of incubation at 495 nm using a plate reader for 8 days (Tecan, Männedorf, Switzerland). Experimental results were standardized to a media only and mock-infected control in each cell line. This experiment was repeated to ensure validity. The ability of CF33-Fluc to replicate in PANC-1, MIA PaCa-2, BxPC-3, SU.86.86, AsPC-1 and Capan-1 was evaluated by a previously described viral growth assay at an MOI of 0.01 for time points 24, 48, and 72 h in duplicate and repeated [11].

### Flow cytometry

PANC-1 and MIA PaCa-2 cells were seeded in 6-well plates and infected next day with CF33 at an MOI of 5 or mock-infected with the virus diluent. Sixteen hours post-infection, the cells were harvested using 5 mM EDTA and washed three times with PBS. Cells were then stained with Alexafluor-488 conjugated anti-calreticulin antibody (ab196158; Abcam, Cambridge, MA) or an isotype antibody (ab199091; Abcam, Cambridge, MA) for 1 h. Stained cells were washed 3 times with FACS buffer, fixed with 4% paraformaldehyde and analyzed on a BD Accuri C6 flow cytometer.

### ATP assays

PANC-1 and MIA PaCa-2 cells were seeded in 6-well plates and infected next day with CF33 at an MOI of 5. Sixteen hours post-infection, supernatants were collected and ATP concentration was measured by the ATP Determination kit (Cat# A22066; Invitrogen, Carlsbad, CA) following manufacturer’s instructions.

### Western blot analysis of high-mobility group box 1 (HMGB1) protein

PANC-1 and MIA PaCa-2 cells were seeded in 6-well plates and infected next day with CF33 at an MOI of 5. Supernatants were collected from infected wells at the indicated time points and concentrated using a column with 3 kDa size cutoff. The concentrated supernatants were loaded (25 μL/well) and ran on a 12% SDS-PAGE. HMGB1 was detected using a rabbit anti-HMGB1 antibody (Cat# ab18256; Abcam, Cambridge, MA) at 1:500 dilution followed by an HRP-labeled goat anti-rabbit secondary antibody (Cat# ab205718; Abcam Cambridge, MA) at 1:5000 dilution.

### Treatment of PANC-1 and MIA PaCa-2 flank xenografts with CF33 and CF33-Fluc

All animal studies were conducted under a City of Hope Institutional Animal Care and Use Committee (IACUC)-approved protocol (IACUC #15003). Forty-one 6-week old Hsd:Athymic Nude-*Foxn1*^*nu*^ female mice *(*Envigo, Indianapolis, IN) were purchased, acclimatized and bilateral flank tumors were generated by injecting 1.25 × 10^6^ PANC-1 (per injection site) in 100 µL PBS containing 50% matrigel. Mice were grouped into three well-matched groups based on their tumor size: PBS injected alone (n = 10), 10^3^ plaque-forming units (PFU) of CF33 (n = 6), or 10^4^ PFU of CF33-Fluc (n = 17). A left-sided intratumoral injection was given when volume of left tumor reached 240 mm^3^ in each mouse. A tenfold higher dose was given in the CF33-Fluc group to ensure visualization on luciferase imaging. Two mice in the PBS group and four mice from the CF33-Fluc treatment group were euthanized on days 3, 13 and 27. Bilateral tumors and organs (heart, lung, liver, spleen, kidney, ovary, brain) were harvested. Tissues were divided into two halves, one half was snap frozen for the purpose of virus titration and the other half was formalin-fixed for immunohistochemical analysis. The remaining mice were euthanized on day 49. In a separate experiment, 8 mice were implanted with 2 × 10^6^ MIA PaCa-2 cells to obtain bilateral flank tumors as described for the PANC-1 model. Mice received a left sided intratumoral injection of PBS alone (n = 3) or 10^5^ PFU of CF33 (n = 5) in 50 µL PBS when tumor volume reached 400 mm^3^. All MIA PaCa-2 xenograft-bearing mice were euthanized at the termination of the experiment on day 43.

For the evaluation of anti-tumor efficacy, tumors were measured twice weekly and tumor volume was calculated using V (mm^3^) = (1/2) × A^2^ × B where A is the shortest and B is the longest diameter. Percent tumor change was calculated from the date of intervention. Virus shedding was determined by performing standard plaque assays on urine, stool and blood samples. Stool and urine from four PANC-1 bearing mice, injected with 10^4^ PFU of CF33-Fluc, was collected twice per week for 1 month post-treatment and blood was collected at the time of euthanasia.

### Luciferase imaging

Firefly luciferin solution was prepared by dissolving 1 g of XenoLight d-luciferin—K^+^ Salt Bioluminescent Substrate (PerkinElmer, Waltham, MA) in 35 mL of PBS. Intraperitoneal delivery was performed in a control mouse and all mice injected with CF33-Fluc and the mice were imaged using Lago X optical imaging system (Spectral Instruments Imaging, Tucson, AZ) after 7 min. Luciferase imaging was performed twice weekly and prior to animal euthanasia.

### Immunohistochemical analysis

Harvested tumors were fixed in formalin for 48 h, paraffin embedded and 5 µm thick sections were obtained. Standard hematoxylin and eosin (H&E) staining was performed. On adjacent tumor sections, immunohistochemical staining was performed. The IHC sections were deparaffinized followed by heat-mediated antigen-retrieval. Briefly, for antigen-retrieval tumor sections were hydrated and steamed for 40 min in IHC-TEK Epitope Retrieval Solution (IHC World, Ellicott City, MD). Following antigen-retrieval, tumor sections were permeabilized with methanol and were blocked using TNB Blocking buffer (PerkinElmer, Waltham, MA) for 20 min. The sections were then incubated with rabbit anti-vaccinia virus antibody diluted 1:100 in TNB blocking buffer (Cat# ab35219; Abcam, Cambridge, MA), overnight in a humidified chamber at 4 °C. The following day, tumor sections were washed and incubated with Alexa Fluor-488-conjugated goat anti-rabbit (Cat# ab150077, Abcam, Cambridge, MA) for 1 h at room temperature. Finally, the sections were counterstained with 4′6-diamidino-2-phenylindole (DAPI) and were imaged using EVOS FL Auto Imaging System (Thermo Fisher Scientific, Waltham, MA).

### Statistical analysis

Statistical analysis was performed using GraphPad Prism (Version 7.01, La Jolla, CA). Comparisons were performed using Student’s t test or one-way ANOVA, when appropriate. Correlation was performed using Pearson’s correlation formula. Logistic or exponential regression line fitting was performed where appropriate. *p* values < 0.05 were considered significant.

## Results

### Chimerization of orthopoxviruses and high-throughput screening

A pool of chimeric orthopoxviruses was generated by co-infecting CV-1 cells with cowpox virus strain Brighton, raccoonpox virus strain Herman, rabbitpox virus strain Utrecht, and VACV strains WR, IHD, Elstree, CL, Lederle-Chorioallantoic and AS at an MOI of 0.01 per virus. Our pilot experiments indicate that CV-1 cells are susceptible to all the orthopoxviruses used in this study. One hundred chimeric orthopoxvirus plaques were picked from CV-1 cells infected with the chimeric orthopoxvirus pool. These 100 plaques were further plaque-purified two more times to yield 100 clonally purified individual chimeric virus isolates.

**Fig. 1:**
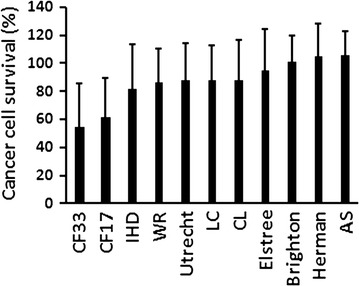
Novel chimeric orthopoxvirus isolates CF33 and CF17 show superior cancer cell killing capability compared to the parental individual wild-type virus strains. Fifty-four solid cancer cell lines in the NCI-60 panel were infected with each virus at an MOI of 0.01. Cell viability was measured at 96 h post infection using MTS assays. Data represent the mean cell survival of 54 cancer cell lines ± sd. CF17 and CF33 demonstrated significantly better cell killing (*p* < 0.001) in the NCI-60 solid tumor cell lines than all nine parental orthopoxvirus strains

Tumor cell-killing activity of 100 chimeric orthopoxvirus isolates, together with nine parental virus strains were evaluated and compared in a panel of the NCI-60 cell lines. Each cell line was infected with each virus at an MOI of 0.01. Cell viability was measured at 96 h post infection using MTS assays. The MOI in this high throughput screening experiment was intentionally kept low, and optimized to compare cell killing in adherent cell lines (the majority of cell lines in the NCI-60 panel are adherent cells) so potent new virus isolates can stand out. This amount of virus, however, was too low to see any significant and consistent cell killing in suspension cell lines. Therefore, the results from six leukemia cell lines were not included in the analysis for the purpose of virus comparison. Among 100 new chimeric orthopoxvirus isolates, isolates CF17 and CF33 demonstrated significantly better cell killing (*p* < 0.001) in the NCI-60 solid tumor cell lines than all nine parental orthopoxvirus strains (Fig. 1), indicating that virus chimerisation can generate a backbone virus that is better than its parental viruses. Both CF17 and CF33 caused significant cell death in the majority of the NCI-60 solid cancer cell lines even at the low MOI of 0.01. CF33 was chosen for further study.

Initial genomic sequence analysis of CF33 revealed that the overall sequence matched more closely to VACV genomes. However, in the absence of published sequences for four out of the nine parental viruses (VACV strains IHD, CL, Lederle-Choriallantoic and AS), we are not able to pinpoint which parts of CF33 came from which parental viruses. Compared to the genomic sequence of VACV strain WR, CF33 contains multiple insertions, deletions and numerous point mutations throughout its genome, thus, it is impossible at this time to pinpoint what sequence variations make the CF33 virus superior to the parental viruses. In future, we plan to perform in-depth sequence analysis for better understanding of the mechanisms through which CF33 out-performs its parental viruses.

### CF33-Fluc infects, replicates in, and kills human pancreatic cancer cell lines in a dose-dependent manner in vitro

**Fig. 2:**
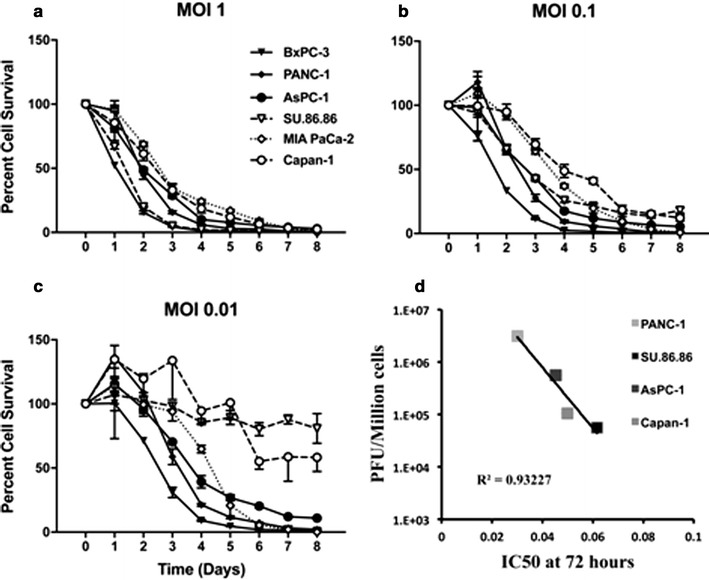
In vitro cytotoxicity and viral replication of chimeric orthopoxvirus CF33-Fluc in pancreatic cancer cell lines. Viability of pancreatic cancer cell lines BxPC-3, PANC-1, MIA PaCa-2, SU.86.86, AsPC-1 and Capan-1 was assessed daily for 8 days after infection with CF33-Fluc. Percent cell survival over time is shown at MOI^†^ = 1 (**a**), MOI = 0.1 (**b**), and MOI 0.01 (**c**). Dose-dependent cell killing was seen in all cell lines. **d** Viral proliferation was inversely correlated to the LD50^‡^ in PANC-1, SU.86.86, AsPC-1 and Capan-1 (R^2^ = 0.932). (^†^MOI, multiplicity of infection. ^‡^LD50, lethal dose for 50% cell killing)

Both CF33 and CF33-Fluc killed all 6 pancreatic cell lines tested in vitro in a dose-dependent manner. Figure 2a–c shows the dose-dependent cancer cell death from CF33-Fluc across all cancer cells tested. Notably, less than 50% pancreatic cancer cell survival occurred across all cell lines at an MOI of 1 by 72 h post-infection. Even at lower MOIs, efficient cell killing was noted in all cell lines. All cell lines, except MIA PaCa-2 and Capan-1, had less than 10% survival by 96 h post-infection at MOI 1. MIA PaCa-2 and Capan-1 had less than 10% survival at 144 h post-infection at MOI 1. All 6 cell lines supported the proliferation of CF33-Fluc at an MOI of 0.01. Most pronounced increases in PFU/million cells were noted in BxPC-3 and PANC-1 cell lines with an approximate 10^4^ fold increase of CF33-Fluc by 2 days post-infection. A correlation of the lethal dose for 50% cell kill (LD50) at 72 h and viral proliferation at 24 h was observed in PANC-1, SU.86.86, AsPC-1, and Capan-1 (R^2^ = 0.932, Fig. 2d).

### CF33 induces immunogenic cell death (ICD) in pancreatic cancer cells

**Fig. 3:**
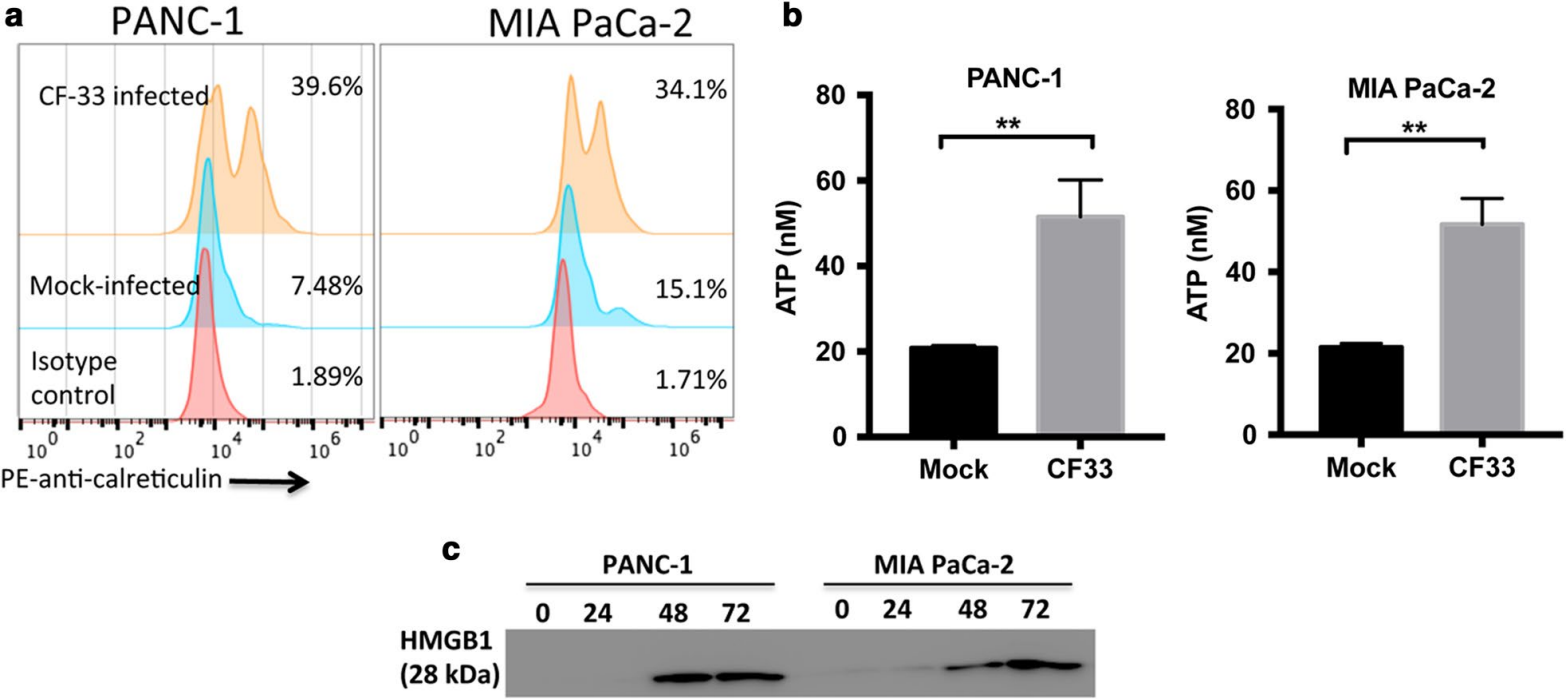
CF33 induces immunogenic cell death in pancreatic cancer cells. PANC-1 and MIA PaCa-2 cells were mock-infected or infected with CF33 at an MOI of 5. Cell surface-exposed CRT was quantified by flow cytometry (**a**), and the release of ATP was measured using ATP assays (**b**) at 16 h post infection. The release of HMGB1 was analyzed by Western blot at 0, 24, 48, and 72 h post infection (**c**). Data represent the mean ± SD, **p < 0.01

Besides direct cell lysis, OVs are known to elicit potent anti-tumoral immune responses, mainly through release of tumor-associated antigens and ICD-related damage-associated molecular patterns (DAMPs) [12]. Calreticulin (CRT), ATP and HMGB1 are critical DAMPs in ICD. Surface-exposed CRT serves as an “eat me” signal for macrophages, neutrophils, and dentritic cells (DCs), whereas ATP acts as a “find me” signal for macrophages and DC precursors. HMGB1 promotes production of cytokines and antigen cross-presentation. Figure 3a shows that infection with CF33 resulted in a five or twofold increase in cell surface-exposed CRT in PANC-1 cells and MIA PaCa-2 cells at 16 h post infection, respectively. At the same time, the release of ATP by infected PANC-1 and MIA PaCa-2 was also significantly increased compared to mock-infected cells (Fig. 3b). The release of HMBG1 became detectable at 48 h post infection for both PANC-1 cells and MIA PaCa-2 cells (Fig. 3c). These results suggested that CF33-induced cell death could be highly immunogenic.

### Low dose of CF33 causes tumor regression without toxicity in PANC-1 and MIA PaCa-2 models in vivo

**Fig. 4:**
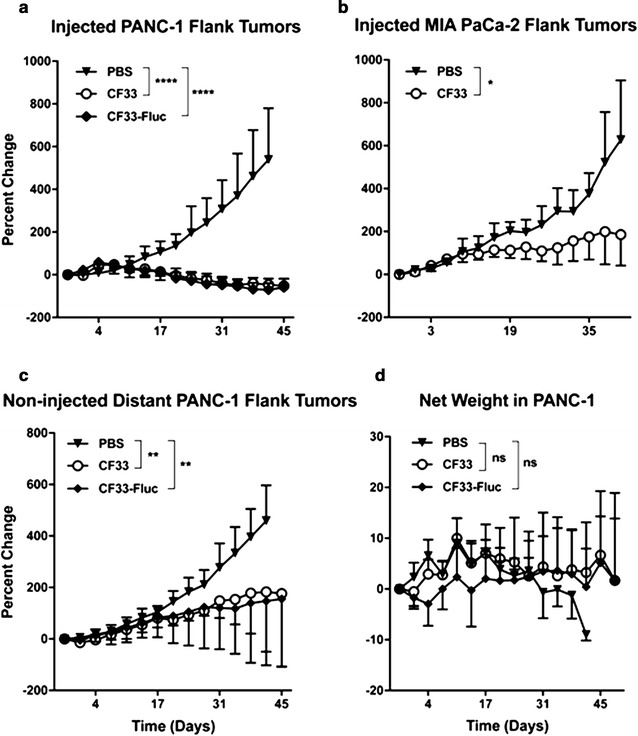
CF33 is safe in mice and causes regression of both injected and non-injected distant tumors at a low dose in PANC-1 and MIA PaCa-2 xenograft models. Female athymic nude mice were implanted in the bilateral flank with either PANC-1 or MIA PaCa-2 and a single injection of 10^3 †^PFU of CF33 for PANC-1 and 10^5^ PFU for MIA PaCa-2 were administered intra-tumorally in the left tumor. **a** Percent change in tumor volume in virus-injected PANC-1 tumors (**a**) and MIA PaCa-2 tumors **b** was significantly lower compared to PBS^‡^-injected tumors. **c** Percent change in the non-injected distant tumors in PANC-1 was significantly smaller with CF33 compared to PBS-injected controls. **d** Toxicity in PANC-1 xenograft bearing mice was determined by percent change in net body weight. No significant difference in weight change was observed between the virus-treated mice and the PBS-treated mice, suggesting that the dose of virus used in this study is safe in immune-compromised mice (*p < 0.05. **p < 0.01. ****p < 0.0001. ns, not significant. ^†^PFU, plaque forming units. ^‡^PBS, phosphate buffered saline)

Significant regression was noted in xenografts injected with intratumoral CF33 at 10^3^ PFU in PANC-1 and 10^5^ PFU in MIA PaCa-2 when compared to PBS injected control (*p* = 0.0001 and *p* = 0.01, respectively, Fig. 4a, b). Toxicity to CF33 and CF33-Fluc was determined by net weight loss. No toxicity was noted in the PANC-1 group injected with a dose of 10^3^ PFU of CF33 or the attenuated CF33-Fluc at 10^4^ PFU when compared to the PBS-injected group (p = 0.25, p = 0.68, respectively, Fig. 4d). PANC-1 bearing mice had no detectable CF33 or CF33-Fluc in the stool, urine or blood at any point of collection.

### CF33-Fluc luciferase activity correlates with tumor regression and spreads to non-injected, distant xenografts in PANC-1

**Fig. 5:**
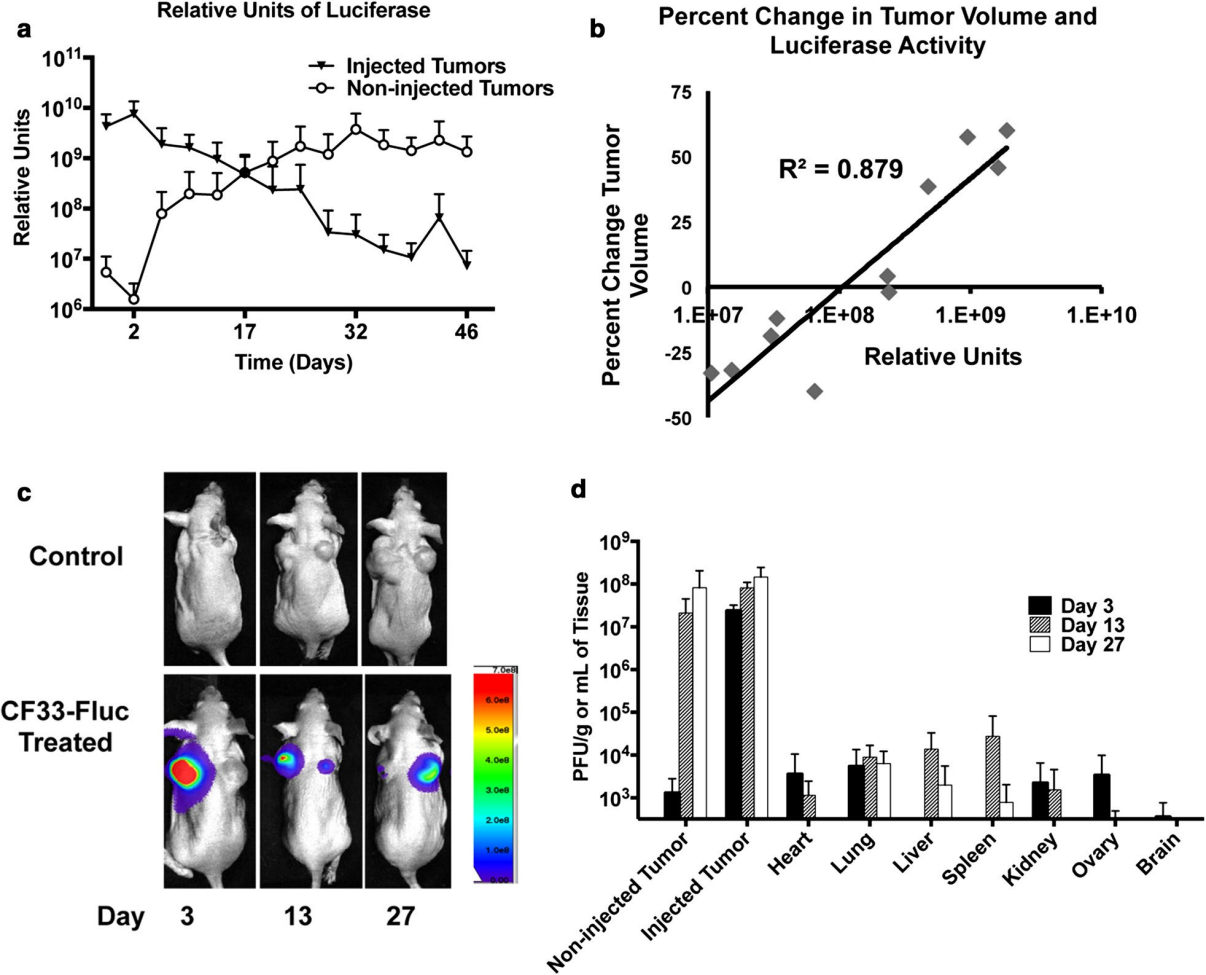
Virus-encoded luciferase activity correlates with tumor regression over time and increasing viral titers in the non-injected tumors. To ensure luciferase visualization, CF33-Fluc was administered at 10^4^ PFU to PANC-1 xenograft-bearing immune-compromised mice. **a** Relative luciferase activity decreased in the injected tumors and increased in the non-injected distant tumor over time. **b** Percent change in CF33-Fluc-injected tumor size and luciferase activity were correlated (R^2^ = 0.879). **c** CF33-Fluc enabled real time in vivo monitoring of viral replication at days 3, 13 and 27 and showed replication in both the injected and non-injected distant tumors. **d** CF33-Fluc titers in the injected tumors are at least 4 logs higher than in other organs, indicating early preferential replication in tumors. Virus titers in the non-injected tumors increased over time, which correlates to luciferase activity

Luciferase signal was noted in the injected tumors at the earliest measured time point of 1 day post-injection (Fig. 5a–c). Decrease in luciferase relative units correlated significantly with percent regression of injected tumor size (R^2^ = 0.879, Fig. 5b). The right sided, or non-injected, distant tumor showed significant regression when compared to the PBS control tumor in both CF33 and CF33-Fluc groups (p = 0.006 and 0.007, respectively, Fig. 4c). Attenuation of the virus did not affect tumor regression in the non-injected tumor as no difference was noted when comparing CF33 or CF33-Fluc (p = 0.99). Luciferase signal was noted in the non-injected tumors after 8 days of treatment and luciferase activity increased in the non-injected tumors for the remainder of the experiment (Fig. 5a, c).

### CF33-Fluc selectively preferentially replicates in PANC-1 xenografts

High CF33-Fluc titers were noted in the injected tumors at 3 days post-treatment (Fig. 5d). Titers above 10^7^ PFU/g tissue were seen in the injected tumors on days 3 and 13. On day 27, the highest titer was noted at 10^8^ PFU/g of injected tumors. The non-injected, distant tumors showed a serial increase in CF33-Fluc titers from 10^3^ PFU/g tissue at day 3 to nearly 10^8^ PFU/g tissue by day 27, which correlated to the increase in luciferase relative units (R^2^ = 0.9972). CF33-Fluc was found in other organs (Fig. 5d). Notably, organs contained at least 10^3^–10^4^ times fewer viruses than the injected tumors. The limit of detection was dependent on organ weight and ranged from 1.2 × 10^2^ to 1.1 × 10^3^ PFU/g.

### Histology and immunohistochemistry shows tumor cell killing and migration of virus from injected to non-injected, distant tumors

**Fig. 6:**
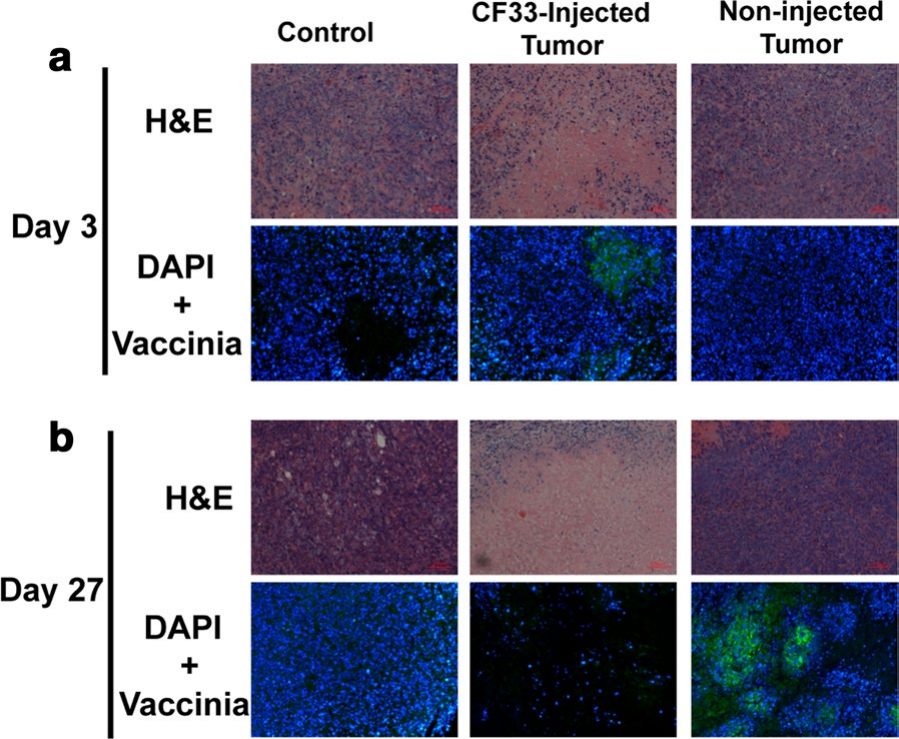
Histology and immunohistochemistry of tumor sections confirm killing of CF33-Fluc injected and non-injected, distant tumors. PBS control, CF33-Fluc-injected and non-injected tumors were harvested at days 3 and 27 and tumor sections were subjected to ^†^H&E or ^‡^IHC staining. **a** Three days following treatment, control tumors show clustered blue nuclei via fluorescent blue ^§^DAPI staining and no evidence of viral infection via fluorescent green anti-viral staining. H&E staining of the injected tumor shows early signs of tumor necrosis and confirms the presence of virus with fluorescent green staining. **b** By day 27, nearly complete necrosis (loss of DAPI staining) is observed in the injected tumor. Green fluorescence confirms the dissemination of CF33-Fluc from the injected to non-injected distant tumors. (^†^H&E, hematoxylin and eosin. ^‡^IHC, immunohistochemistry. ^§^DAPI, 4′6-diamidino-2-phenylindole)

H&E and DAPI fluorescence on sections of the control tumor over time show overgrowth of PANC-1 cells and confirm no viral green fluorescent staining (Fig. 6a). Infection of CF33-Fluc was confirmed at day 3 in the injected tumors through anti-viral green fluorescent staining. H&E shows early signs of tumor necrosis and confirms the presence of virus in only the injected tumor (Fig. 6b). By day 27, H&E showed nearly complete central tumor necrosis in the injected tumor when compared to control and IHC confirmed vaccinia virus in the non-injected, distant tumor. This is consistent with the findings from the luciferase imaging and tumor viral titers.

## Discussion

Pancreatic ductal adenocarcinoma portends a dismal prognosis. Less than 20% of patients are candidates for surgical intervention [13] and of those who are resected, over 80% will ultimately succumb to recurrent disease [14]. Current combination chemotherapeutics in metastatic PDAC have resulted in survival benefits measured in months in the setting of advanced disease [2, 15]. New modalities to treat patients diagnosed with pancreatic cancer are needed. In this paper, we describe the creation and selection of a promising new oncolytic virus CF33. Data supporting CF33 as a potent anti-cancer agent are presented.

Oncolytic viruses are a promising emerging therapy. These viruses selectively infect, replicate in and kill cancer cells. Presently, they are being used in clinical trials to target over 20 types of cancers [16]. Remarkable progress has been made with the first OV, herpes simplex virus 1 (HSV-1) talimogene laherparepvec (T-VEC, Amgen Inc. Thousand Oaks, CA, USA), now approved by the Food and Drug Administration (FDA) after a Phase III trial demonstrated improved response rates in advanced melanoma [17]. Benefits of OVs include tolerable safety profiles that can be further enhanced by virulence gene attenuation [18, 19]. Another benefit is arming the OV to express proteins to enhance the anti-tumor response [20, 21].

Numerous oncolytic viruses have been studied in the preclinical treatment of pancreatic cancer. However, these studies have required high therapeutic doses [22]. In vivo models of PANC-1 xenografts required repeated daily injections of adenovirus at 10^7^–10^8^ PFU in order to achieve tumor regression [23]. Herpes simplex viruses G207 and NV1020 and poxvirus based therapy have also required doses of 10^7^ PFU to cause tumor regression [24, 25]. Typically, in vivo cancer models in athymic mice have required a therapeutic dose of 10^6^–10^8^ PFU for intravenous delivery [22, 26, 27] and at least 10^7^ PFU for intratumoral delivery [28–30]. In our study, in vivo therapeutic efficacy was noted after a single dose of 10^3^ PFU and the mice displayed no toxicity. These results show that CF33 is more potent than previous generations of oncolytic viruses.

This study displays CF33′s unique ability to rapidly kill pancreatic cancer xenografts after a single intratumoral dose in a rapid fashion. In vivo studies of past generations of vaccinia virus in nude mice have described phases of xenograft regression. Initially, tumors increase in size over the first 2 weeks relative to control. The 3rd week consists of a plateau of tumor growth relative to control. Finally, regression in vaccinia-injected tumor volume is observed beyond 3 weeks [22, 31]. With CF33-Fluc, we demonstrated a truncated timeline. The tumors increased in size relative to control by day 4. The plateau occurred from days 4 to 8 and regression, from day 8, onward. This low dose model causing rapid tumor regression again points toward CF33 as a promising candidate for clinical application.

We observed spread of virus to distant tumors. To confirm that the abscopal effect was virally induced, we used luciferase imaging and viral titration data. Although our shedding experiments yielded no detectable virus in the blood, we suspect that viral titers were below the limit of detection in the blood of 5 × 10^2^ PFU/mL. Other modalities of spread are possible; however, low titers detected in other organs would suggest blood-based spreading of virus as seen in other studies [27, 32, 33]. The effect of OV therapy on distant tumors has been studied with Newcastle disease virus (NDV) and was shown to be immune-mediated through an increase in tumor-specific CD4(+) and CD8(+) T-cells [34]. T-VEC has also been studied for the tumor regression in injected and non-injected distant tumors, but again, this is thought to be immune-mediated [35]. As such, clinical trials are ongoing which combine T-VEC with immunomodulators [36]. While our study did not analyze the immune-associated abscopal effect, we believe CF33 is highly oncotropic, and can thereby travel to uninjected tumors and cause direct tumor killing. Future studies involving immune-competent mice will add to the data presented in this study.

While there are further immune-mediated mechanisms of distant tumor killing to be investigated, this study provides evidence for the benefits of direct viral spread. Viral spread may be mediated by the secretion of extracellular enveloped virus (EEV), which is surrounded by a host-derived membrane and released from infected cells [7, 37–39]. Point mutations in the glycoprotein coding A34R gene are known to be associated with increased EEV secretion in the vaccinia strain Western Reserve (WR) [40, 41]. However, CF33 contains the wild-type A34R gene and thus the mechanism of increased spread at this time is under investigation. There are benefits to utilizing the efficient spread to other tumors via intratumoral injection. Improved viral delivery through EEV cloaking enables spread to distant tumors in regionally advanced or metastatic disease. With intravenous delivery, the virus does not yet have the host envelope and therefore encounters innate anti-viral immune responses. OVs in clinical trials often require multiple dosing rounds and therefore therapeutic efficacy is dependent on higher doses or immune evasion [42]. Also, direct delivery to tumors can lower viral toxicity by enhancing preferential intratumoral replication. These factors are important considerations when transitioning to clinical delivery with CF33.

## Conclusions

Through this study we have seen that CF33 and CF33-Fluc are safe and effective in vitro and in vivo. Through chimerization and high throughput screening, we have created a novel virus that requires lower doses to treat pancreatic cancer in a preclinical model. CF33-Fluc replication in non-injected, distant tumors halted tumor growth. Further studies in immune-competent models investigating the mechanisms of rapid tumor regression and viral spread to non-injected tumors following a low viral dose will enhance our understanding of how this novel virus targets and kills pancreatic cancer.

## Abbreviations

ATCC: American Type Culture Collection
CRT: calreticulin
DAMP: damage-associated molecular pattern
DAPI: 4′,6-diamidino-2-phenylindole dihydrochloride
EEV: extracellular enveloped virus
FLuc: firefly luciferase
FOLFIRINOX: folinic acid, fluorouracil, irinotecan hydrochloride, oxaliplatin
H&E: hematoxylin and eosin staining
HMGB1: high-mobility group box 1
ICD: immunogenic cell death
IHC: immunohistochemistry
MOI: multiplicity of infection
OVs: oncolytic viruses
PDAC: pancreatic ductal adenocarcinoma
PFU: plaque forming unit
PBS: phosphate-buffered saline
TK: thymidine kinase

## References

[1] Siegel R, Naishadham D, Jemal A (2013). Cancer statistics, 2013. CA Cancer J Clin.

[2] Conroy T, Desseigne F, Ychou M, Bouche O, Guimbaud R, Becouarn Y, Adenis A, Raoul JL, Gourgou-Bourgade S, de la Fouchardiere C (2011). FOLFIRINOX versus gemcitabine for metastatic pancreatic cancer. N Engl J Med.

[3] Ehrig K, Kilinc MO, Chen NG, Stritzker J, Buckel L, Zhang Q, Szalay AA (2013). Growth inhibition of different human colorectal cancer xenografts after a single intravenous injection of oncolytic vaccinia virus GLV-1h68. J Transl Med.

[4] Haddad D, Zanzonico PB, Carlin S, Chen CH, Chen NG, Zhang Q, Yu YA, Longo V, Mojica K, Aguilar RJ (2012). A vaccinia virus encoding the human sodium iodide symporter facilitates long-term image monitoring of virotherapy and targeted radiotherapy of pancreatic cancer. J Nucl Med.

[5] Parato KA, Breitbach CJ, Le Boeuf F, Wang J, Storbeck C, Ilkow C, Diallo JS, Falls T, Burns J, Garcia V (2012). The oncolytic poxvirus JX-594 selectively replicates in and destroys cancer cells driven by genetic pathways commonly activated in cancers. Mol Ther.

[6] Mallardo M, Leithe E, Schleich S, Roos N, Doglio L, Krijnse Locker J (2002). Relationship between vaccinia virus intracellular cores, early mRNAs, and DNA replication sites. J Virol.

[7] Kirn DH, Wang Y, Liang W, Contag CH, Thorne SH (2008). Enhancing poxvirus oncolytic effects through increased spread and immune evasion. Cancer Res.

[8] Al Yaghchi C, Zhang Z, Alusi G, Lemoine NR, Wang Y (2015). Vaccinia virus, a promising new therapeutic agent for pancreatic cancer. Immunotherapy.

[9] Horton RM, Ho SN, Pullen JK, Hunt HD, Cai Z, Pease LR (1993). Gene splicing by overlap extension. Methods Enzymol.

[10] Falkner FG, Moss B (1990). Transient dominant selection of recombinant vaccinia viruses. J Virol.

[11] Adusumilli PS, Stiles BM, Chan MK, Mullerad M, Eisenberg DP, Ben-Porat L, Huq R, Rusch VW, Fong Y (2006). Imaging and therapy of malignant pleural mesothelioma using replication-competent herpes simplex viruses. J Gene Med.

[12] De Munck J, Binks A, McNeish IA, Aerts JL (2017). Oncolytic virus-induced cell death and immunity: a match made in heaven?. J Leukoc Biol.

[13] Conlon KC, Klimstra DS, Brennan MF (1996). Long-term survival after curative resection for pancreatic ductal adenocarcinoma. Clinicopathologic analysis of 5-year survivors. Ann Surg.

[14] Kleeff J, Reiser C, Hinz U, Bachmann J, Debus J, Jaeger D, Friess H, Buchler MW (2007). Surgery for recurrent pancreatic ductal adenocarcinoma. Ann Surg.

[15] Teague A, Lim KH, Wang-Gillam A (2015). Advanced pancreatic adenocarcinoma: a review of current treatment strategies and developing therapies. Ther Adv Med Oncol.

[16] Choi A, O’Leary M, Fong Y, Chen N (2016). From benchtop to bedside: a review of oncolytic virotherapy. Biomedicines.

[17] Andtbacka RH, Kaufman HL, Collichio F, Amatruda T, Senzer N, Chesney J, Delman KA, Spitler LE, Puzanov I, Agarwala SS (2015). Talimogene laherparepvec improves durable response rate in patients with advanced melanoma. J Clin Oncol.

[18] Heise C, Sampson-Johannes A, Williams A, McCormick F, Von Hoff DD, Kirn DH (1997). ONYX-015, an E1B gene-attenuated adenovirus, causes tumor-specific cytolysis and antitumoral efficacy that can be augmented by standard chemotherapeutic agents. Nat Med.

[19] Buller RM, Smith GL, Cremer K, Notkins AL, Moss B (1985). Decreased virulence of recombinant vaccinia virus expression vectors is associated with a thymidine kinase-negative phenotype. Nature.

[20] Liu BL, Robinson M, Han ZQ, Branston RH, English C, Reay P, McGrath Y, Thomas SK, Thornton M, Bullock P (2003). ICP34.5 deleted herpes simplex virus with enhanced oncolytic, immune stimulating, and anti-tumour properties. Gene Ther.

[21] Liu Z, Ravindranathan R, Kalinski P, Guo ZS, Bartlett DL (2017). Rational combination of oncolytic vaccinia virus and PD-L1 blockade works synergistically to enhance therapeutic efficacy. Nat Commun.

[22] Yu YA, Galanis C, Woo Y, Chen N, Zhang Q, Fong Y, Szalay AA (2009). Regression of human pancreatic tumor xenografts in mice after a single systemic injection of recombinant vaccinia virus GLV-1h68. Mol Cancer Ther.

[23] Motoi F, Sunamura M, Ding L, Duda DG, Yoshida Y, Zhang W, Matsuno S, Hamada H (2000). Effective gene therapy for pancreatic cancer by cytokines mediated by restricted replication-competent adenovirus. Hum Gene Ther.

[24] McAuliffe PF, Jarnagin WR, Johnson P, Delman KA, Federoff H, Fong Y (2000). Effective treatment of pancreatic tumors with two multimutated herpes simplex oncolytic viruses. J Gastrointest Surg.

[25] Tysome JR, Briat A, Alusi G, Cao F, Gao D, Yu J, Wang P, Yang S, Dong Z, Wang S (2009). Lister strain of vaccinia virus armed with endostatin-angiostatin fusion gene as a novel therapeutic agent for human pancreatic cancer. Gene Ther.

[26] Hofmann E, Weibel S, Szalay AA (2014). Combination treatment with oncolytic Vaccinia virus and cyclophosphamide results in synergistic antitumor effects in human lung adenocarcinoma bearing mice. J Transl Med.

[27] Zeh HJ, Bartlett DL (2002). Development of a replication-selective, oncolytic poxvirus for the treatment of human cancers. Cancer Gene Ther.

[28] Vaha-Koskela M, Tahtinen S, Gronberg-Vaha-Koskela S, Taipale K, Saha D, Merisalo-Soikkeli M, Ahonen M, Rouvinen-Lagerstrom N, Hirvinen M, Veckman V (2015). Overcoming tumor resistance by heterologous adeno-poxvirus combination therapy. Mol Ther Oncolytics.

[29] Masuelli L, Fantini M, Benvenuto M, Sacchetti P, Giganti MG, Tresoldi I, Lido P, Lista F, Cavallo F, Nanni P (2014). Intratumoral delivery of recombinant vaccinia virus encoding for ErbB2/Neu inhibits the growth of salivary gland carcinoma cells. J Transl Med.

[30] Kleinpeter P, Fend L, Thioudellet C, Geist M, Sfrontato N, Koerper V, Fahrner C, Schmitt D, Gantzer M, Remy-Ziller C (2016). Vectorization in an oncolytic vaccinia virus of an antibody, a Fab and a scFv against programmed cell death -1 (PD-1) allows their intratumoral delivery and an improved tumor-growth inhibition. Oncoimmunology.

[31] Zhang Q, Yu YA, Wang E, Chen N, Danner RL, Munson PJ, Marincola FM, Szalay AA (2007). Eradication of solid human breast tumors in nude mice with an intravenously injected light-emitting oncolytic vaccinia virus. Cancer Res.

[32] Whitman ED, Tsung K, Paxson J, Norton JA (1994). In vitro and in vivo kinetics of recombinant vaccinia virus cancer-gene therapy. Surgery.

[33] Moss B, Carroll MW, Wyatt LS, Bennink JR, Hirsch VM, Goldstein S, Elkins WR, Fuerst TR, Lifson JD, Piatak M (1996). Host range restricted, non-replicating vaccinia virus vectors as vaccine candidates. Adv Exp Med Biol.

[34] Zamarin D, Holmgaard RB, Subudhi SK, Park JS, Mansour M, Palese P, Merghoub T, Wolchok JD, Allison JP (2014). Localized oncolytic virotherapy overcomes systemic tumor resistance to immune checkpoint blockade immunotherapy. Sci Transl Med.

[35] Senzer NN, Kaufman HL, Amatruda T, Nemunaitis M, Reid T, Daniels G, Gonzalez R, Glaspy J, Whitman E, Harrington K (2009). Phase II clinical trial of a granulocyte-macrophage colony-stimulating factor-encoding, second-generation oncolytic herpesvirus in patients with unresectable metastatic melanoma. J Clin Oncol Off J Am Soc Clin Oncol.

[36] Puzanov I, Milhem MM, Minor D, Hamid O, Li A, Chen L, Chastain M, Gorski KS, Anderson A, Chou J (2016). Talimogene laherparepvec in combination with ipilimumab in previously untreated, unresectable stage IIIB-IV melanoma. J Clin Oncol.

[37] Park BH, Hwang T, Liu TC, Sze DY, Kim JS, Kwon HC, Oh SY, Han SY, Yoon JH, Hong SH (2008). Use of a targeted oncolytic poxvirus, JX-594, in patients with refractory primary or metastatic liver cancer: a phase I trial. Lancet Oncol.

[38] Smith GL, Vanderplasschen A, Law M (2002). The formation and function of extracellular enveloped vaccinia virus. J Gen Virol.

[39] Payne LG (1980). Significance of extracellular enveloped virus in the in vitro and in vivo dissemination of vaccinia. J Gen Virol.

[40] McIntosh AA, Smith GL (1996). Vaccinia virus glycoprotein A34R is required for infectivity of extracellular enveloped virus. J Virol.

[41] Blasco R, Sisler JR, Moss B (1993). Dissociation of progeny vaccinia virus from the cell membrane is regulated by a viral envelope glycoprotein: effect of a point mutation in the lectin homology domain of the A34R gene. J Virol.

[42] Mader EK, Maeyama Y, Lin Y, Butler GW, Russell HM, Galanis E, Russell SJ, Dietz AB, Peng KW (2009). Mesenchymal stem cell carriers protect oncolytic measles viruses from antibody neutralization in an orthotopic ovarian cancer therapy model. Clin Cancer Res.

